# Heparin resistance management during cardiac surgery: a literature review and future directions

**DOI:** 10.1051/ject/2024015

**Published:** 2024-09-20

**Authors:** Salman Pervaiz Butt, Vivek Kakar, Arun Kumar, Nabeel Razzaq, Yasir Saleem, Babar Ali, Nuno Raposo, Fazil Ashiq, Arshad Ghori, Philip Anderson, Nilesh Srivatav, Yazan Aljabery, Salman Abdulaziz, Umer Darr, Gopal Bhatnagar

**Affiliations:** 1 Perfusion Services, Heart Vascular and Thoracic Institute, Cleveland Clinic PO BOX 112412 Abu Dhabi United Arab Emirates; 2 Critical Care Institute, Cleveland Clinic PO BOX 112412 Abu Dhabi United Arab Emirates; 3 Cleveland Clinic Lerner College of Medicine, Western Reserve University 44195 Ohio USA; 4 Anesthesiology Institute, Cleveland Clinic PO BOX 112412 Abu Dhabi United Arab Emirates; 5 Cardiothoracic Surgery Department Heart and Vascular Institute, Cleveland Clinic PO BOX 112412 Abu Dhabi United Arab Emirates; 6 All India Institute of Medical Sciences 110029 New Delhi India; 7 Department of Cardiac Perfusion Technology, Khyber Medical University 25100 Peshawar Pakistan; 8 ECMO Task Force, Department of Health PO BOX 5674 20224 Abu Dhabi United Arab Emirates

**Keywords:** Anticoagulation, Cardiac surgery, Heparin resistance, Activated clotting time, Anti-thrombin

## Abstract

*Introduction*: Heparin, a commonly used anticoagulant in cardiac surgery, binds to antithrombin III (ATIII) to prevent clot formation. However, heparin resistance (HR) can complicate surgical procedures, leading to increased thromboembolic risks and bleeding complications. Proper diagnosis and management of HR are essential for optimizing surgical outcomes. *Methodology*: Diagnosis of HR involves assessing activated clotting time (ACT) and HR assays. Management strategies were identified through a comprehensive review of the literature, including studies exploring heparin dosage adjustments, antithrombin supplementation, and alternative anticoagulants in cardiac surgery patients with HR. A thorough search of relevant studies on HR was conducted using multiple scholarly databases and relevant keywords, resulting in 59 studies that met the inclusion criteria. *Discussion*: HR occurs when patients do not respond adequately to heparin therapy, requiring higher doses or alternative anticoagulants. Mechanisms of HR include AT III deficiency, PF4 interference, and accelerated heparin clearance. Diagnosis involves assessing ACT and HR assays. HR in cardiac surgery can lead to thromboembolic events, increased bleeding, prolonged hospital stays, and elevated healthcare costs. Management strategies include adjusting heparin dosage, supplementing antithrombin levels, and considering alternative anticoagulants. Multidisciplinary management of HR involves collaboration among various specialities. Strategies include additional heparin doses, fresh frozen plasma (FFP) administration, and antithrombin concentrate supplementation. Emerging alternatives to heparin, such as direct thrombin inhibitors and nafamostat mesilate, are also being explored. *Conclusion*: Optimizing the management of HR is crucial for improving surgical outcomes and reducing complications in cardiac surgery patients. Multidisciplinary approaches and emerging anticoagulation strategies hold promise for addressing this challenge effectively.

## Introduction

Antithrombin III (ATIII) is a serine protease inhibitor that inhibits thrombin amongst other factors. Heparin is the most negative biological material known and it is because of this strong negative charge that it exerts its therapeutic effect by binding and activating ATIII through electrostatic interactions, increasing the ATIII-Thrombin reaction by 1000-fold, inhibiting the coagulation cascade [1, 2]. Unfractionated Heparin (UFH) is a commonly used medication for preventing blood clot formation during cardiac surgery, and extracorporeal circulation. Heparin binds to several proteins, but it is the binding to ATIII that is important, as this inactivates thrombin. Binding to ATIII blocks several different clotting factors, importantly Factor IIa and Xa. By inactivating thrombin, it prevents the conversion of fibrinogen to fibrin; this prevents the formation of clots and prolongs the clotting time of blood. Typically, the dosage of UFH for cardiac surgery procedures with cardiopulmonary bypass (CPB) is 300–400 IU/Kg. With this dosage, normally the activated clotting time (ACT) reaches the target within a range of 400–480 s [3].

Anticoagulation is essential in cardiac procedures as the patient’s blood is exposed to foreign surfaces of the heart-lung machine, surgical stress, and room air. This contact triggers the coagulation cascade, leading to life-threatening clotting complications if the patient is not properly anticoagulated. Issues such as consumptive coagulation, excessive postoperative bleeding and thromboembolic events can arise [4].

## Methodology

The methodology involved in diagnosing heparin resistance (HR) includes evaluating ACT and HR assays. To identify management strategies, a comprehensive literature review was carried out, encompassing studies investigating heparin dosage adjustments, antithrombin supplementation, and alternative anticoagulants in cardiac surgery patients with HR. A systematic search of related studies on HR was conducted across various scholarly databases such as Google Scholar, PubMed, and Embase, using specific keywords. The inclusion criteria for selecting studies included relevance to HR management in cardiac surgery patients and publication in peer-reviewed journals. This search yielded 59 studies that satisfied the predetermined inclusion criteria.

## Discussion

### Heparin resistance

Heparin resistance can occur anywhere from 4% to 26% of the time, depending on the first heparin bolus given and the target ACT level needed to start CPB [5]. HR is defined as the inability to achieve a desired ACT or a decreased slope on the Heparin Dose-Response (HDR) curve after adequate heparin dosage. A commonly accepted definition for HR is that >500 U/kg body weight of heparin is required to achieve an ACT of 480 s [6, 7].

The HDR curve aims to account for variability in an individual’s heparin response. Two ACT samples are taken one after a known concentration of heparin and extrapolation from the curve gives a specific concentration of heparin required to achieve a specific ACT. A heparin sensitivity index (HSI) < 1 s/U/kg usually is indicative of HR [4].

Heparin resistance can complicate the continuing management of anticoagulation during and after surgery. To overcome this, the patient may require a higher dose of heparin, alternative anticoagulants, or supplementation with ATIII concentrate to achieve the desired anticoagulant effect. This in turn may pose an increase in the risk of bleeding complications if specific protocols are not in place demonstrating how to deal with this. Additionally, HR may be associated with other underlying factors such as inflammation, genetic variations, or medications, which can further impact surgical outcomes.

Identifying HR before surgery is crucial to optimize anticoagulation strategies. This can be achieved through laboratory tests such as the ACT, or HR assays. By recognizing patients with HR, healthcare providers can adjust the anticoagulation regimen, potentially reducing the risk of complications and improving surgical outcomes

### Mechanisms of heparin resistance

The mechanisms underlying HR are multifaceted and involve ATIII levels, the interaction between heparin and ATIII, and the function of ATIII is mentioned in Tables 1 and 2.

**Table 1:** Illustration of heparin resistance mechanism.

Antithrombin deficiency	
Congenital	Acquired
Reduced levels of AT	Decreased synthesis (e.g., liver disease, malnutrition).
Reduced synthesis and or stability secondary to the gene mutations [15, 16]	Increased clearance (e.g., nephrotic syndrome)
Functionally defective AT	Increased consumption (heparin therapy)
Mutations leading to reduced activity	Upregulated haemostatic system (sepsis, infective endocarditis, DIVC, DVT, PE)
	ECMO, IABP
	Medications (e.g., asparaginase) [17]

**Table 2:** Illustration of heparin resistance diagnosis.

Non-antithrombin mediated
• Increased heparin binding to other proteins, cells and non-endothelial surfaces.
• High platelet count ≥300,000 cells/mm^3^ (due to the activation of PF4, a strong inhibitor of heparin).
• Low albumin concentrations ≤35 g/dL (albumin exhibits heparin-like action).
• Preoperative relative hypovolemia (dehydration leading to increased concentration of other compatible molecules binding to heparin).
• Medications (egg, andexanet Alfa).

Since heparin exerts its effects by catalyzing the anticoagulant activity of ATIII, it has been suggested that antithrombin deficiency is the main cause of HR. Adults’ average ATIII activity ranges from 80% to 120%, and its deficiency is typically described as ATIII activity below 80%. Lemmer et al. looked at ACT levels after heparin induction of >600 IU/kg and found in 53 patients with HR that after administering 500 units of ATIII in 45 patients and 1000 units in 8 patients the mean ACT levels rose from 492 s to 798 s demonstrating the use of ATIII to treat HR during CPB. Although there was an apparent lack of correlation between kaolin ACT levels and ATIII activity noted after >600 IU/kg heparin in 53 patients, this suggests there may be an alternative mechanism present for HR [8].

Assuming ATIII deficiency is the main cause of HR, the reduction can be the result of a congenital deficiency (which has a prevalence of 1 in 3000 people) and these patients tend to have a range of 40–60% of normal [5].

The use of heparin preoperatively 48 h prior contributes to HR, as does enoxaparin. ATIII levels have been seen to decline at approximately 5–7% a day as the thrombin/ATIII complex is cleared via the reticuloendothelial system leading to HR. Although this is still up for debate and may not be clinically significant [6, 7], the exact mechanism is still to be determined and it is thought that it may even be a function of the ACT test when compared with high-dose thrombin test time [8].

Thrombocytosis can also lead to HR as platelet factor 4 (PF4) released from activated platelets binds to UFH, therefore reducing the bioavailability of heparin. PF4 is also a crucial player in heparin-induced thrombocytopenia (HIT), a severe immune complication of heparin therapy. Antibodies to PF4/heparin complexes can develop after heparin exposure and lead to platelet activation, culminating in life-threatening thrombosis [9]. It has been established that PF4 and heparin can form multimolecular complexes, and heparin-induced conformational changes in PF4 render it antigenic, leading to the generation of pathogenic antibodies [9]. These antibodies bind to FcγIIA receptors on platelets, triggering platelet activation and contributing to the prothrombotic state associated with HIT [10].

HR may appear shortly after the onset of thrombocytopenia in these patients. HR in these cases may be due to the neutralization of heparin by PF4 released from activated platelets as previously mentioned. In HIT, a progressive decline in platelet count of more than 50% from baseline or to less than 100,000/μL is typical [11]. Accelerated heparin clearance is another mechanism associated with HR. A study suggested that immune dysregulation in HIT, leading to reduced levels of regulatory cytokines, can contribute to the clearance of heparin and compromise its anticoagulant effect [12]. Temperature also plays a role in heparin clearance. When a procedure is normothermic, the liver metabolic rate is higher, and therefore so is the heparin clearance. In opposition, when deep hypothermia is utilized, the heparin clearance rate is considerably lower [13]. Understanding the factors influencing heparin clearance is vital in optimizing therapeutic strategies for individuals with HR. Heparin resistance can result from increased heparin-binding protein levels, low ATIII levels, increased heparin clearance levels (due to splenomegaly in liver disease), and high factor VIII and fibrinogen levels [12].

A study by Kimura et al. aimed to identify clinical predictors of HR in patients undergoing cardiovascular surgery. It found that 30.7% of the 287 patients experienced HR. Analysis revealed that infective endocarditis (IE), platelet count, and serum fibrinogen and albumin levels were associated with HR. After adjustments for baseline ACT and initial heparin dose, IE (odds ratio 4.57) and albumin levels ≤3.5 g/dL (odds ratio 3.17) were identified as independent predictors of HR. Patients with IE had significantly lower HSI compared to those with other conditions. All patients with HR required additional heparin, and 17 received human antithrombin-III concentrate. The study concluded that infective endocarditis and preoperative hypoalbuminemia are significant independent predictors of HR, indicating a need for further research to optimize anticoagulation strategies for these high-risk patients [14].

Several methods are available to help diagnose HR including ACT, Activated Partial Thromboplastin Time (aPTT), Thromboelastography (TEG), Rotational Thromboelastometry (ROTEM) and Anti-Factor Xa Assay.

One review by Levy et al. discusses the clinical perspectives and management strategies for HR [18]. The authors emphasize that HR should be suspected when higher doses of heparin are required to achieve a therapeutic range of activated partial-thromboplastin time (aPTT), and ACT tests. The chromogenic anti-factor Xa test can also be used to detect UFH function. Furthermore, a study by Bharadwaj et al. explores the occurrence of HR in patients undergoing open-heart surgeries. The authors highlight the importance of achieving therapeutic anticoagulation during procedures like CPB. They emphasize that HR, defined as the inability to achieve therapeutic anticoagulation, has been reported in up to 22% of patients undergoing open-heart surgeries [19]. Another study by Muedra et al. investigates the relationship between antithrombin activity, anticoagulant efficacy of heparin therapy, and perioperative variables in patients undergoing cardiac surgery requiring CPB [20]. The authors explore factors that can influence HR in this surgical population.

Lastly, a review by Warnock et al. provides an overview of heparin’s indications and mechanisms, including its use during cardiac surgery to prevent excess coagulation [21]. The article emphasizes the broad utilization of heparin in hospitals, for various off-label indications, and highlights its effectiveness in preventing thrombotic events during cardiac surgical procedures.

### Consequences of heparin resistance in cardiac surgery

Cardiac surgery involving CPB is a complex procedure that requires effective anticoagulation to prevent thromboembolic events. However, HR, characterized by suboptimal response to heparin therapy, can pose significant challenges during these surgeries. The consequences of HR in cardiac surgery require focusing on the increased risk of thromboembolic events, impaired surgical outcomes, prolonged hospital stay, and elevated healthcare costs.

Chen et al. emphasized the significant interpatient differences in heparin responsiveness, which may result in catastrophic consequences during CPB [4]. Inadequate anticoagulation due to HR can lead to the formation of thrombi, increasing the risk of embolization and subsequent organ damage.

Heparin resistance during cardiac surgery can compromise surgical outcomes, particularly by contributing to increased bleeding and the need for transfusions. Edwards et al. presented case reports highlighting non-antithrombin-mediated HR, which underscored the challenges in achieving adequate anticoagulation for CPB [22]. Suboptimal intraoperative anticoagulation can result in excessive post-operative bleeding, requiring additional transfusions and potentially leading to postoperative complications and prolonged recovery.

A retrospective review aimed at assessing the impact of HR on coronary surgery outcomes revealed that HR was relatively frequent and may impact postoperative morbidity and mortality [23]. Complications arising from inadequate anticoagulation can necessitate extended monitoring, treatment, and recovery periods, ultimately prolonging hospital stays and delaying patient discharge. Heparin resistance in cardiac surgery not only affects patient outcomes but also imposes a financial burden on healthcare systems.

### Management of heparin resistance in cardiac surgery OR

Managing HR necessitates a multidisciplinary approach, involving collaboration among surgeons, anesthesiologists, perfusionists and hematologists. Each speciality brings unique perspectives and expertise to optimize anticoagulation therapy. Effective management of HR is of utmost importance in cardiac surgery, particularly when CPB is involved.

There are multiple pathways for HR patients that allow for the safe commencement of CPB including

- Additional doses of heparin.
- Fresh Frozen Plasma (FFP) administration.
- ATIII AT supplementation via ATIII concentrate.
- Acceptance of a subtherapeutic ACT and commence CPB without additional intervention [5, 24–26].

Heparin dose usually ranges from 300 to 500 U/kg in an attempt to achieve ACT of 480 s and above [27].

A survey-based study explored anticoagulation management and HR during CPB among members of the Society of Cardiovascular Anesthesiologists. It found that 74.9% of the 550 respondents employed empirical weight-based heparin dosing, with most targeting an ACT of 400–480 s to initiate CPB. Despite guidelines recommending higher ACT targets, 17.1% of respondents did not comply, using lower targets or not monitoring heparin effects at all. For HR, which occurs in 4% to 26% of cases, 54.2% used antithrombin concentrates as the first-line treatment [24]. Higher doses of heparin are associated with an increased risk of heparin rebound and postoperative bleeding.

Fresh frozen plasma is concomitantly considered post-heparin therapy for the management of HR. One unit of FFP contains approximately 1 IU of ATIII per ml, and usually 2 units (500 ml) of FFP are administered, to contribute 500 IU of ATIII [8]. Apart from resolving HR, one should be concerned about the transmission of viral infections, volume overload, and the risk of transfusion-related lung injury while administering FFP.

ATIII concentrate has been widely used since 1980 after being considered safe by the Food and Drug Administration (FDA) of the United States. Increasingly, antithrombin concentrates are preferred due to their targeted action and safety profile, offering direct treatment for antithrombin deficiency without the complications associated with FFP. This shift towards specific, less invasive treatments is part of broader clinical practice changes aimed at improving perioperative patient blood management and anticoagulation efficacy [28]. Presently ATIII is available in two forms: the human concentrate (hAT) and recombinant (rAT). Both types of ATIII concentrate are identical and have comparable activity in in vitro thrombin and factor Xa inhibition studies. Each vial of ATIII concentrate contains 500 IU units of ATIII. The usual dose of AT is 1–2 vials, equivalent to 500–1000 IU of ATIII. A formula constituted by Patnik et al. is ATIII dose (IU) = (desired minus current ATIII level as % of normal level) × weight (kg) divided by 1.4 [29]. According to Stammers et al, the average dose of ATIII concentrate required for the treatment of HR is 1,029.0 ±164.5 IU or 14.1 ± 3.4 IU/Kg, when normalized to body weight [26]. A major concern regarding ATIII is cost and availability at certain centres.

A number of studies [30–32] have shown the commencement of CPB in heparin-resistant patients, where the conventional methods failed to reach the desired ACT.

**Figure 1:**
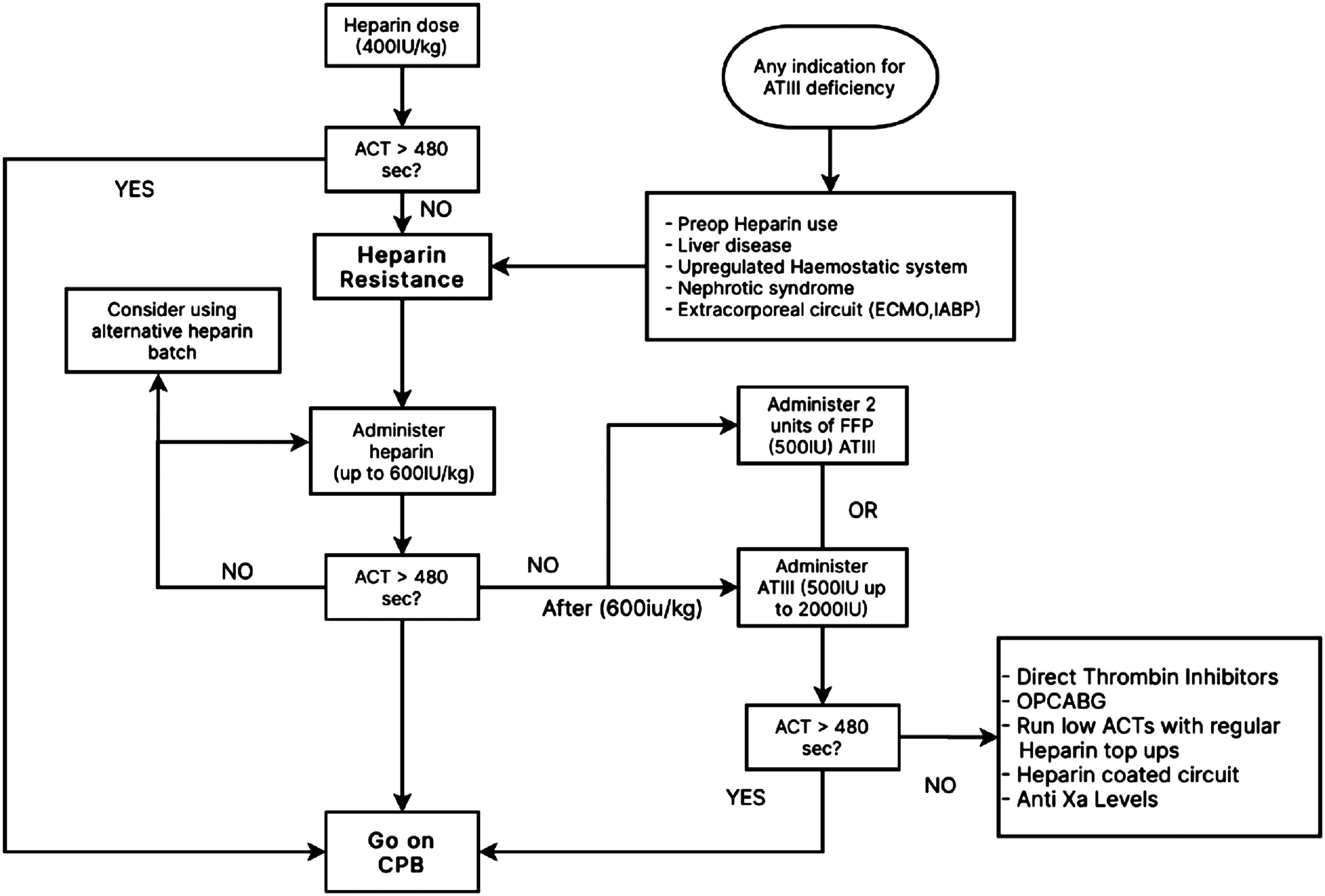
Flow chart showcasing heparin resistance management.

Some critically ill patients are referred for IABP insertion preoperatively, or in some cases, a PCI (Percutaneous coronary intervention) may have been done. Anticoagulation therapy for these procedures is important to prevent thrombosis and embolization. This does not mean HR is a definite but one should be aware it may occur in these patients [33]. Communication of possible HR as a result of the above is essential preoperatively and communication should be fed down to all members of staff in respective departments in an appropriate manner. Staff are then able to prepare for possibilities beforehand. Figure 1 displays a flow chart illustrating the management of HR for CPB commencement.

### Management of heparin resistance in ICU

#### Pre-operative considerations

There are several challenges to the identification of HR in the ICU. First, the use of UFH has largely been replaced with low molecular weight heparin (LMWH) in current practice [34], and the increasing use of direct oral anticoagulants obviates the need to bridge patients initiated on Warfarin with UFH in many cases. Second, even when a continuous infusion of UFH is required in ICU, e.g., acute coronary syndrome, pulmonary embolism, extracorporeal membrane oxygenation, etc., the dose required rarely reaches what has been described as indicative of HR during cardiac surgery (≥300 U/kg) [6, 7]. Third, conventionally, HR is defined based on failure to achieve a set ACT target (400–480 s), an assay that is not routinely used in ICUs around the world as confirmed in the recent International Society of Thrombosis and Hemostasis (ISTH) survey [35]. In 2012, the American College of Chest Physicians described HR in non-surgical patients as failure to achieve therapeutic aPTT despite more than 35,000 U/day of UFH [36]. Following subsequent updates, the topic has been entirely omitted. It is not surprising, therefore, that the ISTH survey revealed significant variability in the definitions of HR used by different centres [35, 37].

Diagnosing HR in the ICU therefore requires a high index of suspicion, and is best done by combining pharmacological, laboratory, and clinical data. We know that several conditions predispose patients to HR. ATIII, which is essential to Heparin response may be low in many ICU patients, either hereditarily or acquired in response to sepsis, disseminated intravascular coagulation, liver diseases or exposure to extracorporeal circuits, e.g., CRRT, ECMO, etc. While ATIII supplementation is a common practice [37] and may improve Heparin response, the level of ATIII that classifies as sufficiently low to cause HR has not been defined. While some platelet count drop can be expected with UFH therapy, when indicated, clinicians must rule out Heparin Induced Thrombocytopaenia which may explain HR [38]. Elevated platelet counts or elevated procoagulant acute phase reactants e.g., fibrinogen, factor VIII, and or von Willebrand factor, in response to sepsis, DIC, CoViD, H1N1, etc. may also lead to HR as measured by aPTT [39].

In patients requiring high doses of UFH, it would be advisable to correlate aPTT against anti-Xa levels. If the anti-Xa is therapeutic despite aPTT being low, then further therapy may be monitored using this assay instead of aPTT. In cases where the anti-Xa are also low, either uses a higher dose of UFH and consider ATIII supplementation if the levels are deemed low, a practice that is not uncommon [40]. In cases where there are clinical indications, e.g., consistent precipitous drop in platelet counts, frequent need of extracorporeal circuit change out, thromboembolic events, etc., HIT must be ruled out.

It may also be reasonable to switch patients requiring high doses of UFH to one of the direct thrombin inhibitors (DTI) e.g., Argatroban or Bivalirudin, that act independently of ATIII, and do not cause immune-mediated thrombocytopaenia. Dosing may need to be adjusted to renal function [Bivalirudin] and hepatic function [Argatroban] and the anticoagulant activity may be monitored using aPTT and or anti-IIa assays. Sadly, there are no approved reversal agents for either of these DTIs, which means patients going for urgent surgeries or procedures must be managed in collaboration with the surgical team, balancing the risks based on indication of anticoagulation and risks of bleeding during the surgery.

#### Post-operative considerations

Patients who are receiving UFH pre-operatively are at a greater risk of having HR intra-operatively and post-operatively [41]. Some case series have shown a significant association between pre-operative UFH use, HR, and fatal myocardial infarction post-coronary artery bypass surgery [42]. High incidence of HR is also noted in patients on pre-operative LMWH and infective endocarditis is also at risk of intra-operative HR [43, 44]. Patients with low pre-operative ATIII and undergoing on-pump cardiac surgery, especially with deep hypothermic circulatory arrest and or requiring post-operative mechanical circulatory support [ECMO, Impella, etc.] are likely to have continuing or worse ATIII deficiency, likely contributing to HR [45].

In patients who were already known to have HR in the pre-operative period and or demonstrated HR in the intra-operative period and require post-operative therapeutic anticoagulation must be managed closely with multidisciplinary involvement, taking into account the urgency of anticoagulation and level of haemostasis following the surgery. Given the feasibility of reliably reversing UFH, it may be advisable to start with UFH, using both aPTT and anti-Xa for monitoring, to reliably reach therapeutic levels and identify HR early. In cases with a high risk of thrombotic complications, e.g., mechanical valves, LVAD, BiVAD, etc., and in cases where the risk of bleeding is deemed sufficiently low, early initiation of oral anticoagulant therapy may be advisable.

### Alternatives for anticoagulation

Direct thrombin inhibitors are an alternative to heparin. Some of the most common are Bivalirudin, Lepirudin and Argatroban. These bind bivalently to thrombin directly, specifically to catalytic and anion-binding exosite of circulating and clot-bound thrombin inhibiting clot formation. The advantage of these over heparin is that they do not require antithrombin to exert their anticoagulant effect and their benefit can also be seen in HIT patients because DTIs do not bind to PF4. The disadvantage however is the lack of a reversal agent leaving the clearance of these drugs from plasma being a combination of renal mechanisms, proteolytic cleavage or liver clearance depending on which DTI is used. This results in them having a half-life impacted by temperature, renal and liver function making the half-life challenging to predict.

There have been multiple studies out there comparing DTI with anticoagulation monitoring. Several laboratory tests are available to monitor DTI activity; aPTT, ACT, thrombin time, dilute thrombin time (DTT) and Ecarin clotting time (ECT) [46–50].

Lepirudin is a DTI which has been shown to be safe. A study by Benoit et al. showed the safe use of Lepirudin with ECT monitoring in a HIT patient on CPB. Whole blood hirudin concentration during CPB was aimed to be above 4 mg/ml^−1^. During the case, 0.1 mg/kg/h lepirudin was given preoperative, 0.2 μg/kg^−1^ bolus just before CPB, and 0.2 μg/kg^−1^ in the priming solution. Complementary boluses of 5 and 10 mg during the procedure were then given according to the ECT. Whole blood hirudin concentration was 3.8–5.8 μg/ml^−1^ with a total lepirudin administration of 44 mg. The case was done successfully and no thrombotic events were observed [51]. Another study done by Greinacher et al. looked at 82 patients with HIT. Eight of these needed CPB where Lepirudin was the anticoagulant and ECT was used for monitoring. An initial bolus of 0.25 mg/kg was given and then subsequently 5 mg boluses as needed when the ECT showed Lepirudin values of <2500 ng/ml. Again, there were no adverse clotting events in any of these patients [52]. These studies show that it is safe to use dosing of 0.5/0.25 mg/kg for lepirudin with 5 mg top-ups at ECT measurements of 2500–4000 ng/ml to run CPB.

In reality, not every hospital has access to ECT testing, for those places there have been case reports where ACT has been used successfully [46, 47, 53, 54]. A study done by Zucker et al. looked at 10 patients. Various ACTs (ACTT(Modified ACT), Celite, Kaolin, ACT+) and ECT levels were investigated against plasma Bivalirudin concentration. Dosing was fixed to (1.0 mg/kg bolus followed by a 2.5 mg/kg/h infusion for all patients. The ACTT and the ECT showed greater sensitivity to bivalirudin (∼28.5 s/μg/ml bivalirudin) compared with the other ACTs evaluated (∼14 s/μg/ml), this was especially true at low concentrations of bivalirudin (<10 μg/ml), with the ECT and ACTT showing slopes near 40, and the ACT slopes varying from 18 to 27 sec/microg/ml. Although ACTs were still sensitive to Bivalirudin concentration [53].

Another case study done was by Boysan et al. using Bivalirudin during CPB. They used the same 1.0 mg/kg bolus followed by a 2.5 mg/kg/h infusion. Top-ups of 0.5 mg/kg were added as necessary and the patient had no thrombotic events using ACT, their ACT levels were always above 300 s but often did not reach 400. As a result, they decided to stop renal clearance of Bivalirudin to aid the ACT [46].

Nikolaides also did a case report using Bivalirudin. The same dosing strategies as above were initially implemented but they later found with their 100 kg patient this dose was not enough to raise the ACT as required so they gave a total of 250 mg of Bivalirudin as a loading dose and then increased their infusion to 5 mg/kg/h. Using this method with ACT was successful and the case was completed without any adverse effects. This shows that anticoagulation management should be considered patient specific and the dosing should not be blindly followed for every patient. Anticoagulation is multifaceted and there would be an increase in risk and safety without looking at Anticoagulation monitoring indicators [47].

Nafamostat mesilate (NM) is a synthetic protease inhibitor which has been shown to inhibit factor XII, fibrinolysis, platelet aggregation, and blood-foreign surface interaction. It has been used previously in open heart surgery and reduced bleeding [55]. NM is another drug which has been used in conjunction with Heparin in heparin-resistant patients undergoing CPB. A study done by Kikura et al. looked at 870 cardiac surgery patients, 190 of which had HR, these received a bolus of NM 10–20 mg plus 25–50 mg/h of NM with 100 u/kg of intravenous heparin every 1.5–2 h to maintain ACTs of > 480 s. Ischemic strokes were only found in 1 patient (0.5%) in patients receiving NM as opposed to 10 patients (1.5%) in patients without [56]. Other studies have shown successful CPB cases using the same combination of NM and LMWH in infective endocarditis patients with a high risk of cerebral bleeds [5, 57–59]. More studies will have to be done to find out if this strategy is a good alternative treatment in HR patients and may potentially be safe to use routinely in CPB.

## Conclusion

HR during cardiac surgery poses significant risks, leading to adverse outcomes. Advances in understanding its mechanisms have paved the way for new anticoagulation strategies. While antithrombin deficiency is a primary cause, factors like platelet activation and altered fibrinogen levels also play a role. Understanding these mechanisms is crucial for overcoming HR during CPB in cardiac surgery.

Emerging strategies to address HR include supplementing antithrombin levels to enhance heparin’s effect and using direct thrombin inhibitors. Further research is needed to identify novel mechanisms and potential biomarkers for personalized anticoagulation approaches.HR challenges cardiac surgery by increasing thromboembolic risks, impairing surgical outcomes, prolonging hospital stays, and raising healthcare costs. Diagnosis involves methods like ACT and HR assays. Effective management requires adjusting heparin dosage, supplementing antithrombin levels, and considering alternative anticoagulants. Optimizing HR management is crucial for improving surgical outcomes and reducing complications in cardiac surgery patients.

## Data Availability

The research data are available on request from the authors.
